# Aromatic Dipeptide Homologue-Based Hydrogels for Photocontrolled Drug Release

**DOI:** 10.3390/nano12101643

**Published:** 2022-05-11

**Authors:** Chloé Guilbaud-Chéreau, Bhimareddy Dinesh, Laurène Wagner, Olivier Chaloin, Cécilia Ménard-Moyon, Alberto Bianco

**Affiliations:** CNRS, Immunology, Immunopathology and Therapeutic Chemistry, UPR 3572, University of Strasbourg, ISIS, 67000 Strasbourg, France; chloe.guilb@gmail.com (C.G.-C.); dineshb@gmx.com (B.D.); laurene.wagner@univ-lorraine.fr (L.W.); o.chaloin@ibmc-cnrs.unistra.fr (O.C.)

**Keywords:** carbon nanotubes, graphene oxide, phenylalanine, tyrosine, self-assembly

## Abstract

Peptide-based hydrogels are considered of special importance due to their biocompatibility and biodegradability. They have a wide range of applications in the biomedical field, such as drug delivery, tissue engineering, wound healing, cell culture media, and biosensing. Nevertheless, peptide-based hydrogels composed of natural α-amino acids are limited for in vivo applications because of the possible degradation by proteolytic enzymes. To circumvent this issue, the incorporation of extra methylene groups within the peptide sequence and the protection of the terminal amino group can increase the enzymatic stability. In this context, we investigated the self-assembly capacity of aromatic dipeptides (Boc-α-diphenylalanine and Boc-α-dityrosine) and their β- and γ-homologues and developed stable hydrogels. Surprisingly, only the Boc-diphenylalanine analogues were able to self-assemble and form hydrogels. A model drug, l-ascorbic acid, and oxidized carbon nanotubes (CNTs) or graphene oxide were then incorporated into the hydrogels. Under near-infrared light irradiation, the photothermal effect of the carbon nanomaterials induced the destabilization of the gel structure, which caused the release of a high amount of drug, thus providing opportunities for photocontrolled on-demand drug release.

## 1. Introduction

Short peptide-based hydrogels have drawn tremendous interest in biomaterial research [1,2,3,4]. These types of hydrogels have been extensively explored in recent years for tissue engineering, as scaffolds for wound healing, and for drug release [5,6,7]. Similar to amino acids, the self-assembly of peptides in aqueous solution to form hydrogels is controlled by a perfect balance between hydrophobicity and hydrophilicity. Aromatic dipeptides can self-assemble through π-π interactions, leading to the formation of nanotubes [8] or hollow spherical nanostructures [9]. Diphenylalanine, which is the core recognition of β-amyloid peptide found in Alzheimer’s disease, was demonstrated to be the simplest building block leading to the formation of highly ordered nanostructures such as nanovesicles or nanotubes [10,11]. Gazit and coworkers have described the thermal and chemical stability of diphenylalanine nanotubes both in aqueous solution and dry conditions, revealing high resilience at elevated temperatures [12]. Within the class of aromatic peptides, fluorenylmethyloxycarbonyl (Fmoc)-protected dipeptides can spontaneously form fibrous networks [13]. In this regard, aromatic interactions between peptides can play a key role in the gelation process [14,15] and N-terminus protected aromatic groups, such as Fmoc, naphthyl, and pyrenyl, which favor the formation of hydrogels [16,17,18]. For example, Fmoc- or naphthyl-protected dipeptides are able to generate hydrogels in the presence of glucono-δ-lactone, whose hydrolysis to gluconic acid induces a pH change and triggers the gelation [19]. The self-assembly properties of *tert*-butoxycarbonyl (Boc)-protected peptides have also been studied, for example, for their antibacterial properties [20]. Recently, Ravarino et al. prepared a transparent hydrogel, obtained by the self-assembly of a Boc-protected dipeptide made by the combination of 3,4-difluorophenylalanine and 4-methyl-5-carboxy-oxazolidin-2-one, which relies on the contribution of halogen bonds provided by fluorine atoms [21].

The majority of peptide-based hydrogels are composed of natural α-amino acid residues and present some limitations for in vivo applications related to their possible degradation by proteolytic enzymes. Therefore, there is an imperative need for the development of proteolytically stable hydrogels. The incorporation of extra methylene groups into the peptide backbone and the protection of the terminal amino acid can play an important role in the stability against peptidases [22,23]. A previous study performed by our team concerning the self-assembly of Boc-diphenylalanine backbone homologues with functionalized carbon nanotubes (CNTs) demonstrated that β- and γ-homologues self-assemble in water to generate fibers of different dimensions and shapes [24]. In particular, β- and γ-homologues of Boc-diphenylalanine were able to generate pH-sensitive fibers in 1:1 ethanol/water solution, with morphological modifications at acidic pH. This behavior provides the basis for the development of novel pH-sensitive β- and γ-peptide-based materials, including drug delivery systems. In addition, the fibrillar nature of the β- and γ-diphenylalanine peptides in water suggests their use to design novel hydrogels. We have also shown that the β- and γ-diphenylalanine peptides conjugated to carbon nanotubes could self-assemble into fibrillar dendritic structures, favor the growth of neuronal cells, and modulate neuronal functions [25]. Moreover, we previously reported that l-tyrosine can form well-ordered assemblies with different morphologies, such as nanoribbons, fibers, or branched structures [26]. Dityrosine peptide is also able to form nanofibers in a buffer solution and therefore can be exploited as a low-molecular-weight gelator [27].

Carbon nanomaterials, including CNTs and graphene-family nanomaterials, offer many advantages, not only for neuronal applications [25], but also as photothermal agents, thanks to their capacity to absorb near-infrared (NIR) light and convert it into heat. Carbon nanotubes (CNTs) are rolled-up graphene sheets of single-layer carbon atoms. They can be single-walled CNTs with a diameter of 1 nm or multi-walled CNTs, made of several nanotubes, with a diameter up to 100 nm. Graphene sheets consist of sp^2^-hybridized carbon atoms arranged in a hexagon lattice. Both functionalized carbon nanotubes and graphene oxide are widely used for biomedical applications. The absence of toxicity in vitro and in vivo of functionalized CNTs and GO has been demonstrated by our group and others [28,29,30,31,32]. Their photothermal properties have been exploited to treat tumors or destroy bacteria [33,34]. The incorporation of carbon nanomaterials into hydrogels can be used to precisely control the release of therapeutic molecules, thanks to an enhanced local temperature under NIR light irradiation inducing the destructuration of the supramolecular gel structures [35].

In this work, we studied the self-assembly properties of Boc-α-diphenylalanine and Boc-α-dityrosine, as well as their β- and γ-homologues under different conditions, and their capacity to form hydrogels. A model drug, l-ascorbic acid, and oxidized CNTs (ox-CNTs) or graphene oxide (GO) were incorporated into the hydrogels. The characterization of the hydrogels was performed using transmission electron microscopy (TEM) and circular dichroism (CD). Finally, the drug release capacity of the gels was investigated under NIR light irradiation.

## 2. Materials and Methods

### 2.1. Materials

Boc-amino acid derivatives were purchased from Iris Biotech GmbH (Marktredwitz, Germany), and the solvent from Carlo Erba reagent (Peypin, France). Boc-γ-Tyr(OBzl)-OH was prepared as described by Smrcina et al. [36]. Boc-β^3^(*R*)Phe-β^3^(*R*)Phe-OH and Boc-γ^4^(*R*)Phe-γ^4^(*R*)Phe-OH were synthesized following a protocol previously reported [24]. Pristine multi-walled carbon nanotubes (CNTs) (20–30 nm diameter, 0.5–2 μm length, 95% purity; batch 1240XH) were purchased from Nanostructured and Amorphous Materials, Inc. (Katy, TX, USA). CNTs were oxidized following a previously reported protocol [24]. GO was obtained from Grupo Antolin (Burgos, Spain). All other reagents were purchased from different commercial suppliers and used as received. The sonication was performed in an ultrasonic bath Elmasonic P (100 W, 37 kHz). An 808 nm laser diode system from Roithner Lasertechnik (LOS-BLD-0808-2W-C/P) and a thermal imaging camera from FLIR ONE were used for the photothermal studies. The centrifugation was done with a centrifuge 5415 R from Eppendorf. HPLC was performed using a Nucleosil 100-5 Waters C_18_ reverse phase HPLC column and a Waters Alliance e2695 separation module. The column was used with a 1.2 mL·min^−1^ flow rate of a gradient from 0 to 100% of B (A = H_2_O/0.1% TFA; B = CH_3_CN/0.08% TFA) for 20 min.

### 2.2. Methods

*Transmission electron microscopy.* TEM analysis was performed with a Hitachi 7500 transmission electron microscope (Hitachi High Technologies Corporation, Tokyo, Japan) with an accelerative voltage of 80 kV equipped with an AMT Hamamatsu digital camera (Hamamatsu Photonics, Hamamatsu City, Japan). To prepare the TEM samples, 10 µL of each hydrogel was deposited onto a carbon-coated copper grid (Formvar/Carbon 300 mesh; Cu from Delta Microscopies). The gel was allowed to stand for 1–2 min, after which the sample was removed by capillarity using a filter paper. The grid was allowed to dry under ambient condition.

*Circular dichroism.* The dipeptide gels were formed using the pH-switch method. After the addition of HCl, the samples were transferred into a 0.05 mm path length quartz cuvette. CD spectra were recorded on a JASCO J-810 spectropolarimeter at room temperature from 200–350 nm with 1.0 nm step, a scanning speed of 100 nm·min^−1^, and 1-s integration time.

*Photothermal studies.* The heating profiles were obtained by monitoring the temperature increase during exposure to 808 nm laser with a power of 2 W·cm^−2^ for 10 min. Each photothermal measurement was repeated three times. The maximum temperatures and infrared thermographic maps were recorded by an infrared thermal imaging camera.

### 2.3. Synthesis of Boc-Dipeptides

Boc-α(*S*)Tyr-α(*S*)Tyr-OH (**1**)

*Boc-Tyr(OBzl)-Tyr-OBzl (**1a**).* To a solution of Boc-Tyr(OBzl)-OSu (5.87 g, 12.54 mmol) in CH_3_CN (50 mL), H-Tyr-OBzl (5.1 g, 18.82 mmol) dissolved in water (50 mL) was added. The reaction mixture was stirred for 8 h at room temperature. Acetonitrile was removed in vacuo, and the residue was dissolved in ethyl acetate (100 mL). The organic layer was washed twice by a saturated bicarbonate solution, 1 M potassium hydrogen sulfate, brine, dried over sodium sulfate and concentrated in vacuo to produce a solid. Yield: 51% (2 g), purity by RP-HPLC: ≥97%, LC/MS: 624.98.

*Boc-Tyr-Tyr-OH (**1**).* Boc-Tyr(OBzl)-Tyr-OBzl **1a** (2 g, 3.2 mmol) was hydrogenated at room temperature in 1,4-dioxane (50 mL) in the presence of a 10% Pd/C catalyst. After 24 h, the catalyst was removed by filtration and the filtrate concentrated in vacuo to leave a residue that solidified upon trituration in a mixture of cyclohexane/diethyl ether. The solid was collected, washed with diethyl ether, and dried in vacuo to produce the Boc-α-dipeptide (**1**). Yield: 94% (1.33 g), purity by RP-HPLC: ≥97%, LC/MS: 444.72.

Boc-β^3^(*R*)Tyr-β^3^(*R*)Tyr-OH (**2**)

*Boc-β-Tyr(OBzl)-β-Tyr(OBzl)-OMe (**2a**).* Boc-β-Tyr(OBzl)-OH (0.50 g, 1.3 mmol) was dissolved in DMF (10 mL) containing H-(β)Tyr(OBzl)-OMe (0.39 g, 1.3 mmol), benzotriazol-1-yloxytris(dimethylamino)phosphonium hexafluorophosphate (BOP) (0.58 g, 1.3 mmol), and *N*,*N*-diisopropylethylamine (0.51 mL, 3 mmol). After 4 h at room temperature, CHCl_3_ (100 mL) was added. The organic layer was washed twice by a saturated bicarbonate solution, 1 M potassium hydrogen sulfate, brine, dried over sodium sulfate and concentrated in vacuo to produce a white solid. Yield: 94% (0.8 g), purity by RP-HPLC: ≥95%, LC/MS: 666.54.

*Boc-β-Tyr(OBzl)-β-Tyr(OBzl)-OH (**2b**).* Boc-β-Tyr(OBzl)-β-Tyr(OBzl)-OMe **2a** (0.8 g, 1.2 mmol) was partially deprotected by a mixture of NaOH 1 N (2.4 mL, 2.4 mmol) and tetrahydrofuran (10 mL). After 4 h at room temperature, the mixture was concentrated *in vacuo* to leave a residue that precipitated upon trituration in diethyl ether/cyclohexane (1:1). Yield: 88% (0.7 g), purity by RP-HPLC: ≥95%.

*Boc-β-Tyr-β-Tyr-OH (**2**).* Boc-β-Tyr(OBzl)-β-Tyr(OBzl)-OH **2b** (0.7 g, 1.1 mmol) was hydrogenated at room temperature in methanol (30 mL) in the presence of a 10% Pd/C catalyst. After 18 h, the catalyst was removed by filtration and the filtrate was concentrated in vacuo to leave a residue that solidified upon trituration in a mixture of diisopropyl ether/diethyl ether. The solid was collected, washed with diisopropyl ether, and dried in vacuo to give the Boc-β-dipeptide (**2**). Yield: 94% (0.48 g), purity by RP-HPLC: ≥97%, LC/MS: 472.82.

Boc-γ^4^(*R*)Tyr-γ^4^(*R*)Tyr-OH (**3**)

*Boc-γ-Tyr(OBzl)-γ-Tyr(OBzl)-OMe (**3a**).* To a solution of Boc-γ-Tyr(OBzl)-OH (1.0 g, 2.5 mmol) in MeOH (10 mL), SOCl_2_ (907 µL, 5 mmol) was added dropwise at 0 °C. After 1 h, the mixture was concentrated in vacuo to leave a residue that precipitated upon trituration in diethyl ether. Yield: 96% (0.75 g), purity by RP-HPLC: ≥95%.

Boc-γ-Tyr(OBzl)-OH (0.96 g, 2.4 mmol) was dissolved in DMF (8 mL) containing γ-Tyr(OBzl)-OMe (0.75 g, 2.4 mmol), BOP (1.06 g, 2.4 mmol), and *N*,*N*-diisopropylethylamine (0.85 mL, 5 mmol). After 3 h at room temperature, ethyl acetate (100 mL) was added. The organic layer was washed twice by a saturated bicarbonate solution, 1 M potassium hydrogen sulfate, brine, dried over sodium sulfate, and concentrated in vacuo to produce a solid. Yield: 88% (1.47 g), purity by RP-HPLC: ≥95%, LC/MS: 695.07.

*Boc-γ-Tyr(OBzl)-γ-Tyr(OBzl)-OH (**3b**).* Boc-γ-Tyr(OBzl)-γ-Tyr(OBzl)-OMe **3a** (1.47 g, 2.11 mmol) was partially deprotected by a mixture of NaOH 1 N (4.1 mL, 4.1 mmol) and tetrahydrofuran (20 mL). After 18 h at room temperature, the mixture was concentrated in vacuo to leave a residue that precipitated upon trituration in diethyl ether. Yield: 100% (1.43 g), Purity by RP-HPLC: ≥95%, LC/MS: 681.01.

*Boc-γ-Tyr-γ-Tyr-OH (**3**).* Boc-γ-Tyr(OBzl)-γ-Tyr(OBzl)-OH **3b** (1.43 g, 2.11 mmol) was hydrogenated at room temperature in methanol (30 mL) in the presence of a 10% Pd/C catalyst. After 18 h, the catalyst was removed by filtration and the filtrate concentrated in vacuo to leave a residue that precipitated upon trituration in diethyl ether. The solid was washed with diisopropyl ether and dried in vacuo to produce the Boc-γ-dipeptide (**3**). Yield: 95% (1.0 g), Purity by RP-HPLC: ≥97%, LC/MS: 501.00.

Boc-α(*S*)Phe-α(S)Phe-OH (**4**)

*H-α-Phe-OMe (**4a**).* Boc-α-Phe-OH (1 g, 3.8 mmol) was dissolved in dry methanol (20 mL) in ice, and SOCl_2_ (0.5 mL, 7.6 mmol) was slowly dropped. The reaction was stirred under cold conditions for 20 min and then at room temperature. After 3 h, the solvent was concentrated in vacuo to produce a gummy solid, which upon trituration in diethyl ether, was dried in vacuo to produce pure H-*α*-Phe-OMe (**4a**). The crude product was directly used for further coupling by confirming the deprotection of Boc and methyl ester formation by LC/MS: 179.10.

*Boc-α-Phe-α*-*Phe-OMe (**4b**).* Boc-α-Phe-OH (1 g, 3.8 mmol) dissolved in CH_2_Cl_2_ (20 mL) was activated by 1-[3-(dimethylamino)propyl]-3-ethylcarbodiimide hydrochloride (0.8 g, 4.2 mmol) and *N*-hydroxysuccinimide (0.5 g, 4.2 mmol). The reaction mixture was stirred at 0 °C for 30 min; H-Phe-OMe (0.82 g, 3.8 mmol) was added, and the reaction mixture was stirred at room temperature. After 4 h, CH_2_Cl_2_ (150 mL) was added. The organic layer was washed twice by a saturated bicarbonate solution, 1 M potassium hydrogen sulfate, brine, dried over sodium sulfate, and concentrated in vacuo to give a white solid. Yield: 95% (1.5 g), purity by RP-HPLC: ≥95%, LC/MS: 426.50.

*Boc-α-Phe-α-Phe-OH (**4**).* Boc-α-Phe-α-Phe-OMe **4a** (1.5 g, 3.5 mmol) was deprotected by a mixture of NaOH 1 N (5 mL, 5 mmol) and tetrahydrofuran (15 mL). After 4 h at room temperature, the mixture was concentrated in vacuo to leave a residue that precipitated upon trituration in diethyl ether/cyclohexane (1:1) and dried in vacuo to produce the Boc-α-Phe-α-Phe-OH (4b). Yield: 82% (1.2 g), purity by RP-HPLC: ≥95%, LC/MS: 412.49.

### 2.4. Self-Assembly Protocol

To observe the self-assembly process of the dipeptides in water, the samples were dissolved in MilliQ^®^ water at a concentration of 0.5 mg·mL^−1^. Samples were sonicated 10 s in a water bath and left for 1 h before deposition of 10 µL on a TEM grid, followed by evaporation of the drop at room temperature.

### 2.5. Gel Preparation

*Gelation using the solvent-triggered method.* For the hydrogel preparation the dipeptide Boc-α-Phe-α-Phe-OH, Boc-β^3^(*R*)Phe-β^3^(*R*)Phe-OH or Boc-γ^4^(*R*)Phe-γ^4^(*R*)Phe-OH was first dissolved in an organic solvent to improve the solubility (DMSO, HFIP, or MeOH) at a concentration of 247 mM via 10 s sonication to help the dissolution process. Then, the solution was diluted using MilliQ^®^ water to induce the gelation with a final concentration of 2.45, 4.9, or 9.8 mM in 2%, 5% or 10% organic solvent/water (*v*/*v*) and left under ambient conditions. The gelation of the samples was confirmed by the vial inversion test to observe if the hydrogels were stable (Figure S1).

*Gelation using the pH-switch method.* The dipeptide was dissolved in a solution of 10 mM NaOH to reach a final concentration of 4.9 mM. Stirring and sonication were used to help the dissolution process. To turn the solution from transparent to opaque, a minimum amount of 0.5 M HCl was added (1% (*v*/*v*) for Boc-α-Phe-α-Phe-OH and Boc-γ^4^(*R*)Phe-γ^4^(*R*)Phe-OH, 7% (*v*/*v*) for Boc-β^3^(*R*)Phe-β^3^(*R*)Phe-OH). After gentle stirring, the samples were left under ambient conditions. The gelation was confirmed by the vial inversion test to observe if the hydrogels were stable.

### 2.6. Incorporation of Oxidized CNTs or GO

GO or ox-CNTs were incorporated in the hydrogels prepared using the pH-switch method. The carbon nanomaterials were added directly to the dipeptides previously dissolved in NaOH solution with a final concentration of 255 µg/mL^−1^ of ox-CNTs or GO. Both materials were dispersed, in turn, using water bath sonication for 10 min for ox-CNTs and 3 min for GO.

### 2.7. Drug Loading

The Boc-diphenylalanine analogue was dissolved in 200 µL of 10 mM NaOH at a final concentration of 4.9 mM. Stirring and sonication helped the dissolution process. Then, ox-CNTs or GO were incorporated into the solution and dispersed by sonication at a final concentration of the nanomaterials of 0.025 wt%. The drug (l-ascorbic acid) was incorporated at a final concentration of 0.7 mg·mL^−1^ and 0.5 M HCl was added to trigger the gelation.

### 2.8. Release of l-Ascorbic Acid

The Boc-diphenylalanine analogue was dissolved in 10 mM NaOH at a final concentration of 4.9 mM. Stirring and sonication helped the dissolution process. Then, ox-CNTs or GO were incorporated into the gels and dispersed by sonication (final concentration of the nanomaterials: 0.025 wt%). Finally, l-ascorbic acid was incorporated at a final concentration of 0.7 mg·mL^−1^, and HCl 0.5 M was added to trigger the gelation. NIR light irradiation (808 nm, 3 cm distance of the top of the gels, 2 W·cm^−2^ for 10 min) was applied to the gels. The volume of released water was withdrawn, centrifuged (12300 rpm, 10 min), and the amount of l-ascorbic acid was assessed by HPLC.

## 3. Results and Discussion

### 3.1. Peptide Self-Assembly in Water

**Figure 1:**
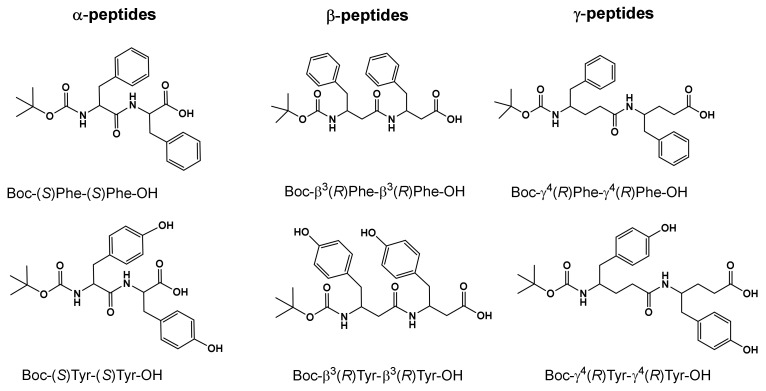
Molecular structures of the Boc-diphenylalanine and Boc-dityrosine analogues used in this study.

**Figure 2:**
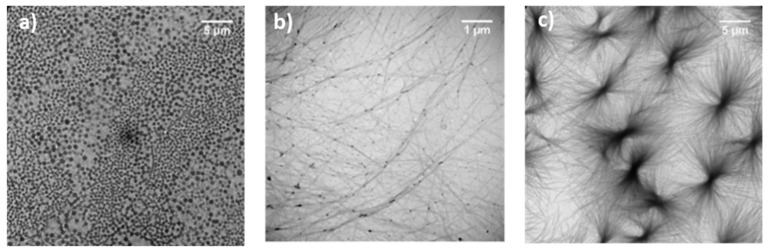
TEM images of (**a**) Boc-α(*S*)Phe-α(*S*)Phe-OH, (**b**) Boc-β^3^(*R*)Phe-β^3^(*R*)Phe-OH, and (**c**) Boc-γ^4^(*R*)Phe-γ^4^(*R*)Phe-OH in water at a concentration of 0.5 mg·mL^−1^.

We initially studied the self-assembly properties of Boc-diphenylalanine and Boc-dityrosine analogues in water. The β- and γ-analogues of Boc-diphenylalanine were synthesized as previously reported [24], while the preparation of the Boc-dityrosine peptides and the Boc-α-diphenylalanine is described in the Supplementary Materials (Figure 1). For the self-assembly experiments, the dipeptides were dissolved in water at a concentration of 0.5 mg·mL^−1^, and they were allowed to self-assemble for 1 h (Figure 2). TEM images showed that Boc-α(*S*)Phe-α(*S*)Phe-OH could self-assemble into nanospheres (Figure 2a), whereas Boc-β^3^(*R*)Phe-β^3^(*R*)Phe-OH and Boc-γ^4^(*R*)Phe-γ^4^(*R*)Phe-OH formed fibers (Figure 2b,c). The nanospheres obtained with Boc-α(*S*)Phe-α(*S*)Phe-OH were variable in size with a diameter between 200 and 700 nm. Similarly, spheres with a diameter around 1 µm were previously obtained by dissolving the same dipeptide in hexafluoroisopropanol (HFIP) at a concentration of 100 mg·mL^−1^ and subsequently diluting the solution in ethanol [37,38]. Boc-β^3^(*R*)Phe-β^3^(*R*)Phe-OH self-assembled instead in highly oriented fibers, while Boc-γ^4^(*R*)Phe-γ^4^(*R*)Phe-OH revealed a dense network of fibers growing from a central nucleation point, as we already demonstrated in a former study [24]. The difference in structure induced by the introduction of the additional carbons within the framework, from α to γ-dipeptides, is likely due to the increased flexibility within the backbone. By increasing the length of the carbon chain, the peptides tend to align by supramolecular interactions to form fibers, contrary to Boc-α(*S*)Phe-α(*S*)Phe-OH, which tends to aggregate into spheres. A previous study performed by our group on the supramolecular assembly of phenylalanine derivatives showed that the self-assembly was mainly based on aromatic interactions (π−π and CH−π interactions) [39].

We studied the self-assembly capacity of Boc-dityrosine analogues under the same conditions. Unfortunately, the dityrosine peptides did not assemble into well-organized structures and we only observed aggregates on the TEM grids (data not shown). In the literature, examples of short sequences of unprotected dityrosine and trityrosine able to form long nanofibers in aqueous solutions have been reported, whereas tetratyrosine tended to bundle randomly [27]. These results indicate that the presence of the Boc group likely inhibited the fiber growth. The hydroxyl group of the tyrosine seemed also to play a key role in preventing the self-assembly process and the formation of the fibers.

### 3.2. Formation of the Hydrogels

To induce the formation of hydrogels, we first tested the solvent-triggered method that we previously exploited to form stimuli-responsive hydrogels using protected amino acids [39]. For this purpose, the dipeptides were first dissolved in DMSO at a concentration of 247 mM and then diluted in water to reach a final concentration of 4.9 mM in 2% DMSO in water. The gelation of the different samples was confirmed by the vial inversion test [39]. A homogeneous gel was obtained using Boc-γ^4^(*R*)Phe-γ^4^(*R*)Phe-OH in less than 5 min, while a heterogeneous gel containing aggregates and a viscous solution were observed with Boc-β^3^(*R*)Phe-β^3^(*R*)Phe-OH in 13 h and with Boc-α(*S*)Phe-α(*S*)Phe-OH, respectively. Different parameters were modified to optimize the gelation protocol, including temperature, pH, dipeptide concentration, nature and volume of the organic solvent, and the presence of monovalent or divalent salts (Table 1). The modification of the temperature from room temperature down to 4 °C or up to 37 °C allowed the formation of a stable and homogeneous gel for Boc-α(*S*)Phe-α(*S*)Phe-OH in 15 h only at 37 °C, whereas heterogeneous gels were obtained using Boc-β^3^(*R*)Phe-β^3^(*R*)Phe-OH at the two temperatures with no difference in time of gelation. The formation of homogeneous gels with Boc-γ^4^(*R*)Phe-γ^4^(*R*)Phe-OH was not affected by the temperature. Examples of α-diphenylalanine involved in the formation of hydrogels have reported that this peptide requires combination with other molecules (e.g., short polyethylene glycol chains) [40] to generate stable gels, while Fmoc-α(*S*)Phe-α(*S*)Phe-OH alone can form hydrogels only when dissolved in HFIP [41]. To our knowledge, no examples of hydrogels have been reported for the β- and γ-homologues.

Regarding the influence of the pH, we observed that Boc-α(*S*)Phe-α(*S*)Phe-OH formed a heterogeneous gel in 15 h, but this was not stable over time, and no gels were formed with the β- and γ-homologues when a basic aqueous solution (pH 11) was added. Under acidic conditions (pH 4), Boc-α(*S*)Phe-α(*S*)Phe-OH was also capable of forming a heterogeneous gel in only 2 h, but this was not stable over time, whereas the β- and γ-dipeptides generated stable heterogeneous and homogenous gels, in 24 h and less than 5, min respectively (Figure S2). In our previous study, the dipeptides showed morphological modifications of the self-assembled nanostructures obtained at different pH values. From neutral to acidic conditions, we observed a transformation of the fibers into partially coalesced spheres in the case of Boc-β^3^(*R*)Phe-β^3^(*R*)Phe-OH, while the fibers disassembled forming an irregular film in the case of Boc-γ^4^(*R*)Phe-γ^4^(*R*)Phe-OH [24]. These results are in agreement with the pH-sensitivity of the three systems.

The change in DMSO with HFIP or methanol did not give positive results, as no gels were obtained in the case of HFIP for the three peptides (Figure S3), while the formation of a heterogeneous gel was observed only for Boc-β^3^(*R*)Phe-β^3^(*R*)Phe-OH in the presence of MeOH after 24 h. Therefore, the nature of the organic solvent plays an important role on the gelation properties of the diphenylalanine analogues. Observing that the β-dipeptide was poorly soluble in 2% DMSO in water, we increased the percentage of DMSO to 5 and 10%. A stable gel was obtained using 10% DMSO in water in 5 min, but with some water release during the gel formation (Figure S4). Nevertheless, this method was not chosen for further experiments as the amount of DMSO may lead to undesired toxic effects on cells and tissues [42].

It was previously reported that the use of salts, including NaCl, can accelerate the formation of supramolecular nanostructures of Fmoc-dipeptides [43]. Therefore, we tested the incorporation of NaCl or CaCl_2_ at 10 mM, 25 mM and 100 mM to induce gelation. These experiments were not conclusive, as they showed the formation of aggregates (Figure S5).

Finally, the influence of the dipeptide concentration was studied using half or double quantity of peptides in 2% DMSO/H_2_O. Interestingly, a gel was obtained for Boc-α(*S*)Phe-α(*S*)Phe-OH at a concentration of 2.45 mM. Nevertheless, the hydrogel was not stable beyond two days. When the concentration was 9.8 mM, only Boc-γ^4^(*R*)Phe-γ^4^(*R*)Phe-OH formed a homogeneous gel in a few minutes. Overall, the optimization of the different parameters in the solvent-triggered approach did not allow us to obtain a unique protocol to produce homogeneous and stable gels for the three dipeptide analogues.

As the dipeptides have a free COOH, which can be deprotonated under pH modification, another method based on pH changes (called pH-switch) was tested [44]. This protocol consisted of dissolving the dipeptides at a concentration of 4.9 mM in a basic solution and triggering the gel formation by acidification. The dipeptide analogues were quickly solubilized in a 10 mM NaOH solution under sonication for a short time, followed by the dropwise addition of 0.5 M HCl solution. We were delighted to observe complete gelation of the three Boc-diphenylalanine analogues using the pH-switch method. We observed a color modification of the solutions from transparent to opaque after the addition of an appropriate volume of HCl (1% (*v*/*v*) for Boc-α(*S*)Phe-α(*S*)Phe-OH and Boc-γ^4^(*R*)Phe-γ^4^(*R*)Phe-OH and 7% (*v*/*v*) for Boc-β^3^(*R*)Phe-β^3^(*R*)Phe-OH). The Boc-β^3^(*R*)Phe-β^3^(*R*)Phe-OH and Boc-γ^4^(*R*)Phe-γ^4^(*R*)Phe-OH hydrogels formed instantaneously and in approximatively 1 min and 1 h, respectively. In contrast, the gelation of Boc-α(*S*)Phe-α(*S*)Phe-OH occurred after about 7 h, and this gel was less stable over time compared to the other two hydrogels. A structural degradation and water release were observed after one day.

With this pH-switch method, we were therefore able to prepare hydrogels with the three Boc-diphenylalanine analogues, and we observed a higher stability of the gels with the addition of one (beta) or two (gamma) methylene groups within the dipeptide backbone. The instability of the Boc-α(*S*)Phe-α(*S*)Phe-OH hydrogel can be correlated to the self-assembly capacity of this dipeptide. Indeed, Boc-α(*S*)Phe-α(*S*)Phe-OH self-assembles into spheres in aqueous media (Figure 2a), in contrast with the Boc-β^3^(*R*)Phe-β^3^(*R*)Phe-OH and Boc-γ^4^(*R*)Phe-γ^4^(*R*)Phe-OH homologues forming fibers (Figure 2b,c).

Following the successful gelation of the Boc-diphenylalanine analogues, we aimed to compare the gelation properties of these peptides to those of the Boc-dityrosine analogues. In this regard, the hydrogelation protocols tested for the Boc-diphenylalanine analogues were also applied to Boc-α(*S*)Tyr-α(*S*)Tyr-OH, Boc-β^3^(*R*)Tyr-β^3^(*R*)Tyr-OH, and Boc-γ^4^(*R*)Tyr-γ^4^(*R*)Tyr-OH. Unfortunately, the Boc-dityrosine analogues did not form hydrogels under any of the different conditions including the pH-switch method. The formation of fibers in water is generally essential to obtain hydrogels by allowing the creation of a network encapsulating water molecules. As mentioned above, the three Boc-dityrosine analogues did not self-assemble in water, contrary to Boc-β^3^(*R*)Phe-β^3^(*R*)Phe-OH and Boc-γ^4^(*R*)Phe-γ^4^(*R*)Phe-OH that formed long fibers. This result indicates that the hydroxyl group present in the tyrosine side chain has a key role in inhibiting the self-assembly into nanofibers, leading to gelation. For this reason, the following experiments focused only on the Boc-diphenylalanine peptides due to the absence of hydrogelation observed with the Boc-dityrosine analogues.

### 3.3. Incorporation of Carbon Nanomaterials and Photothermal Properties

**Figure 3:**
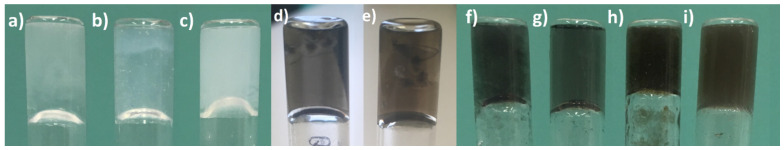
Photographs of the hydrogels obtained by applying the pH-switch method. (**a**) Boc-α(*S*)Phe-α(*S*)Phe-OH, (**b**) Boc-β^3^(*R*)Phe-β^3^(*R*)Phe-OH, (**c**) Boc-γ^4^(*R*)Phe-γ^4^(*R*)Phe-OH, (**d**) Boc-α(*S*)Phe-α(*S*)Phe-OH + 0.025 wt% ox-CNTs, (**e**) Boc-α(*S*)Phe-α(*S*)Phe-OH + 0.025 wt% GO, (**f**) Boc-β^3^(*R*)Phe-β^3^(*R*)Phe-OH + 0.025 wt% ox-CNTs, (**g**) Boc-γ^4^(*R*)Phe-γ^4^(*R*)Phe-OH + 0.025 wt% ox-CNTs, (**h**) Boc-β^3^(*R*)Phe-β^3^(*R*)Phe-OH + 0.025 wt% GO, and (**i**) Boc-γ^4^(*R*)Phe-γ^4^(*R*)Phe-OH + 0.025 wt% GO.

Considering the excellent photothermal properties of carbon nanomaterials under NIR light irradiation [33], we decided to develop Boc-diphenylalanine hybrid hydrogels containing ox-CNTs or GO for phototriggered drug release applications [39]. To prepare these hydrogels, suspensions of ox-CNTs and GO at a concentration of 0.025 wt% were added to the basic dipeptide solutions, followed by the addition of HCl. The gelation process was not impacted by the incorporation of the carbon nanomaterials, and the gelation time remained identical for the three Boc-diphenylalanine analogues (Figure 3). While GO and ox-CNTs aggregated in Boc-α(*S*)Phe-α(*S*)Phe-OH and Boc-β^3^(*R*)Phe-β^3^(*R*)Phe-OH gels, both nanomaterials were well dispersed in the Boc-γ^4^(*R*)Phe-γ^4^(*R*)Phe-OH hydrogel. The higher amount of acid added for the hydrogelation of Boc-β^3^(*R*)Phe-β^3^(*R*)Phe-OH likely induced some aggregation of ox-CNTs and GO.

**Figure 4:**
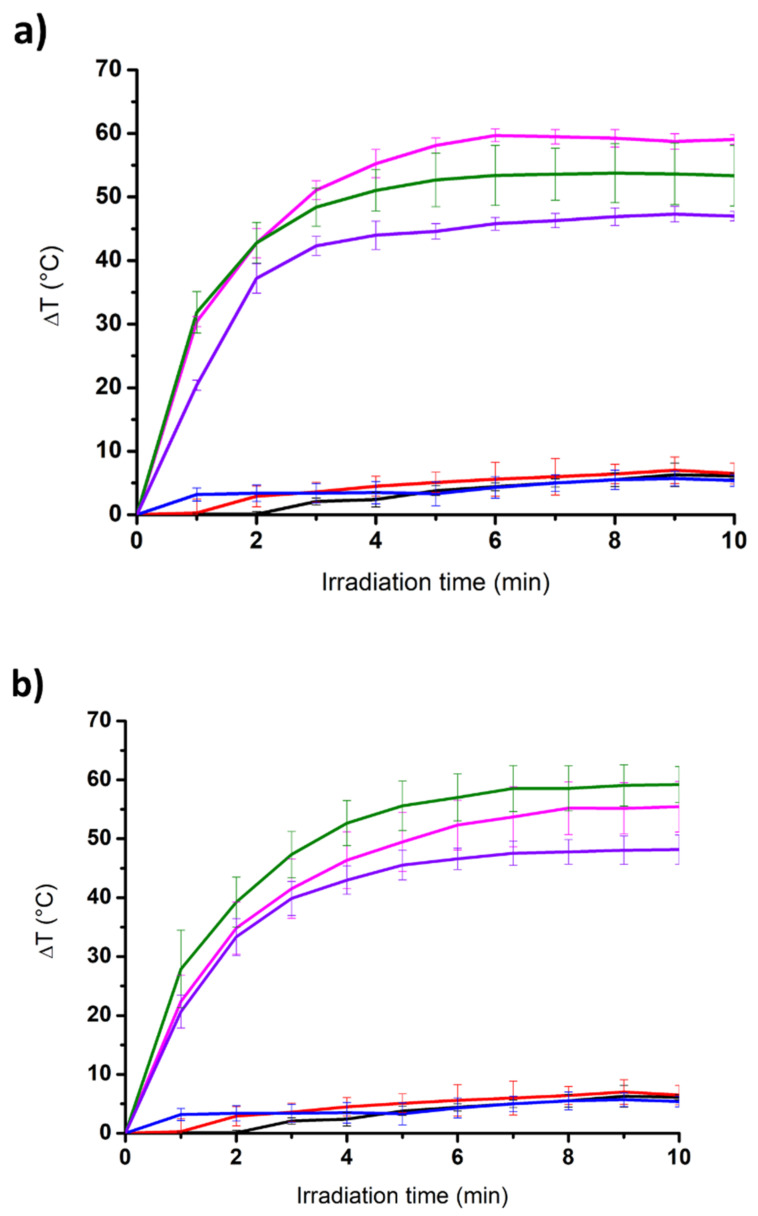
Temperature increase of the native and hybrid gels in the presence of (**a**) ox-CNTs and (**b**) GO when exposed to an NIR laser (808 nm, 2 W·cm^−2^) as a function of the laser irradiation time. Black = Boc-α(*S*)Phe-α(*S*)Phe-OH, red = of Boc-β^3^(*R*)Phe-β^3^(*R*)Phe-OH, blue = Boc-γ^4^(*R*)Phe-γ^4^(*R*)Phe-OH, magenta = Boc-α(*S*)Phe-α(*S*)Phe-OH + 0.025 wt% carbon nanomaterial, green = Boc-β^3^(*R*)Phe-β^3^(*R*)Phe-OH + 0.025 wt% carbon nanomaterial, violet = Boc-γ^4^(*R*)Phe-γ^4^(*R*)Phe-OH + 0.025 wt% carbon nanomaterial.

We studied the photothermal properties of the dipeptide-based hydrogels under NIR light irradiation using a laser emitting at 808 nm (Figure 4) [45]. As expected, the change in temperature for the three native gels (i.e., devoid of carbon nanomaterials) was very low with an increase of 6 °C, 6.5 °C, and 5.4 °C after 10 min of irradiation for Boc-α(*S*)Phe-α(*S*)Phe-OH, Boc-β^3^(*R*)Phe-β^3^(*R*)Phe-OH, and Boc-γ^4^(*R*)Phe-γ^4^(*R*)Phe-OH, respectively. The three gels remained totally intact after irradiation. On the contrary, all of the hybrid gels were degraded under NIR light irradiation. The hybrid gel Boc-α(*S*)Phe-α(*S*)Phe-OH was completely liquefied, whereas the irradiation of the hybrid gels Boc-β^3^(*R*)Phe-β^3^(*R*)Phe-OH and Boc-γ^4^(*R*)Phe-γ^4^(*R*)Phe-OH led to the appearance of a liquid phase together with a remaining gel block. An increase in the temperature in the range of 47.0 °C to 59.2 °C was observed after 10 min. The highest temperature increase (50–60 °C) was obtained for the hybrid gels Boc-α(*S*)Phe-α(*S*)Phe-OH and its β-homologue. The lowest temperature increase was obtained for the hybrid gels Boc-γ^4^(*R*)Phe-γ^4^(*R*)Phe-OH with a temperature increase slightly below 50 °C.

### 3.4. Morphological Study

**Figure 5:**
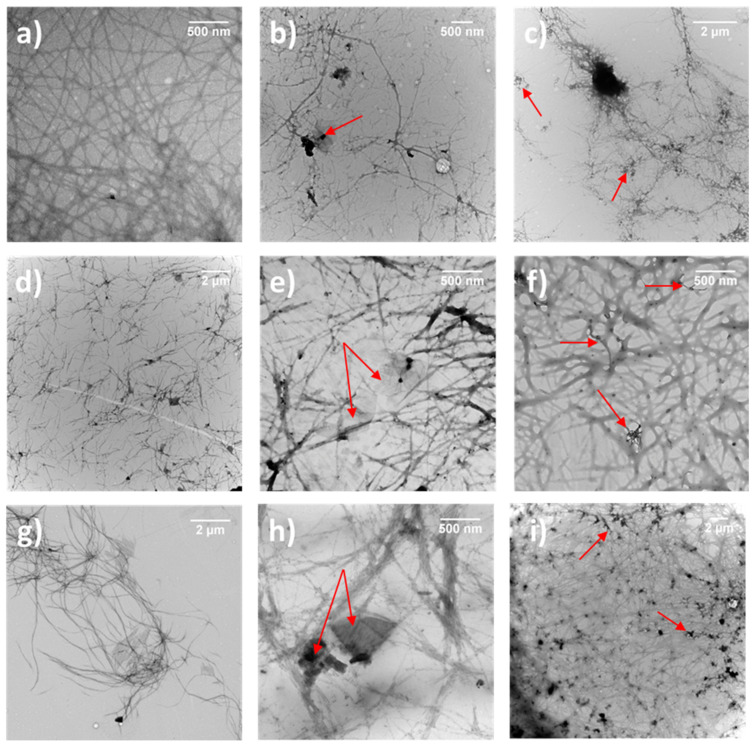
TEM images of (**a**) Boc-α(*S*)Phe-α(*S*)Phe-OH, (**b**) Boc-α(*S*)Phe-α(*S*)Phe-OH + 0.025 wt% GO, (**c**) Boc-α(*S*)Phe-α(*S*)Phe-OH + 0.025 wt% ox-CNTs, (**d**) Boc-β^3^(*R*)Phe-β^3^(*R*)Phe-OH, (**e**) Boc-β^3^(*R*)Phe-β^3^(*R*)Phe-OH + 0.025 wt% GO, (**f**) Boc-β^3^(*R*)Phe-β^3^(*R*)Phe-OH + 0.025 wt% ox-CNTs, (**g**) Boc-γ^4^(*R*)Phe-γ^4^(*R*)Phe-OH, (**h**) Boc-γ^4^(*R*)Phe-γ^4^(*R*)Phe-OH + 0.025 wt% GO, and (**i**) Boc-γ^4^(*R*)Phe-γ^4^(*R*)Phe-OH + 0.025 wt% ox-CNTs. The arrows show GO sheets and ox-CNTs.

The morphological characterization of the different native and hybrid hydrogels formed using the pH-switch method was performed by TEM (Figure 5). Whereas Boc-α(*S*)Phe-α(*S*)Phe-OH self-assembled in spheres in water (Figure 2a), we instead observed long fibers with a width of 10–30 nm in the gel state (Figure 5a and Figure S6a). The presence of ox-CNTs or GO into the hydrogels had no impact on the diameter of the fibers (Figure 5b,c and Figure S6d,g). The other two gels containing Boc-β^3^(*R*)Phe-β^3^(*R*)Phe-OH and Boc-γ^4^(*R*)Phe-γ^4^(*R*)Phe-OH were also constituted of long interconnected fibers with a larger diameter distribution in the range of 20–90 nm (Figure 5d,g and Figure S6b,c). The presence of ox-CNTs or GO did not disturb the self-assembly of both dipeptides into fibers of rather similar size (Figure 5e,f,h,i and Figure S6e,f,h,i). The ox-CNTs and GO were in close contact with the fibers, which is certainly due to π-π interactions with the aromatic moieties of the dipeptides, similar to our previous study using protected aromatic amino acids [39].

### 3.5. Conformational Studies

**Figure 6:**
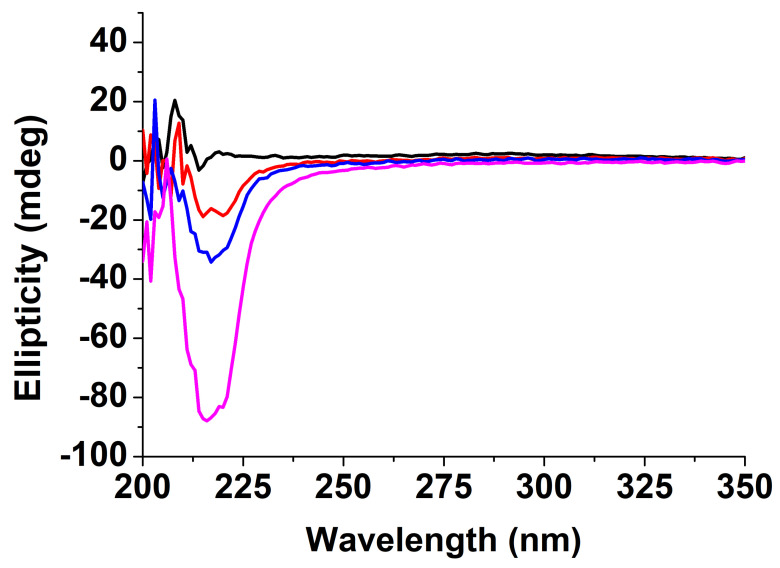
CD spectra of the gel Boc-α(*S*)Phe-α(*S*)Phe-OH (black = directly after the addition of HCl into the cuvette at t0, red = after 1 h, blue = 2 h, and magenta = 20 h).

**Figure 7:**
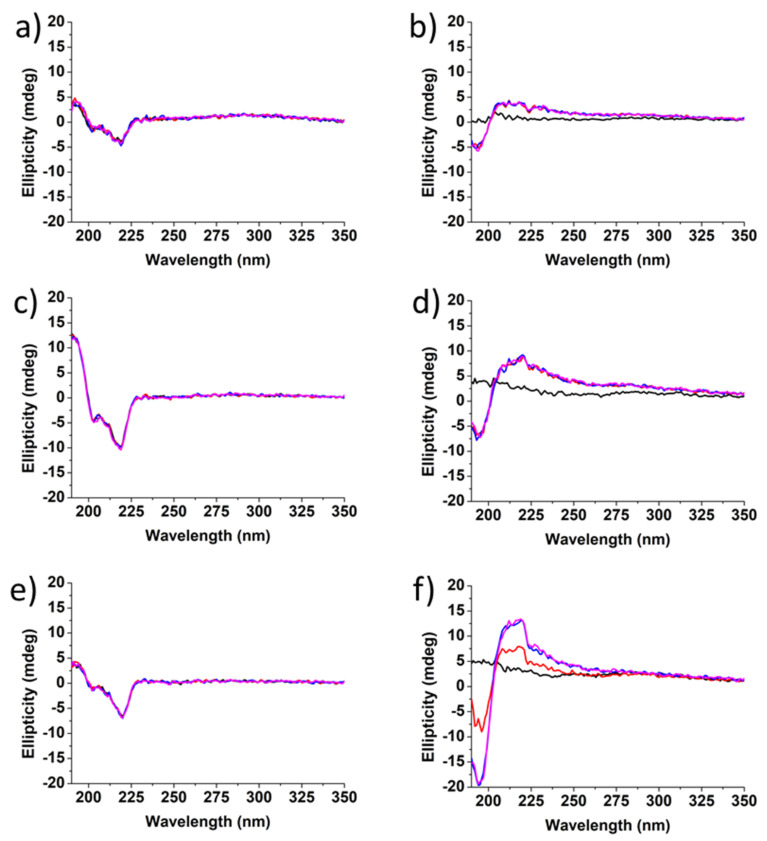
CD spectra of (**a**) Boc-β^3^(*R*)Phe-β^3^(*R*)Phe-OH; (**b**) Boc-γ^4^(*R*)Phe-γ^4^(*R*)Phe-OH; (**c**) Boc-β^3^(*R*)Phe-β^3^(*R*)Phe-OH + 0.025 wt% ox-CNTs; (**d**) Boc-γ^4^(*R*)Phe-γ^4^(*R*)Phe-OH + 0.025 wt% ox-CNTs; (**e**) Boc-β^3^(*R*)Phe-β^3^(*R*)Phe-OH + 0.025 wt% GO; (**f**) Boc-γ^4^(*R*)Phe-γ^4^(*R*)Phe-OH + 0.025 wt% GO (black = directly after the addition of HCl into the cuvette at t0, red = after 10 min, blue = after 20 min, magenta = 30 min).

The structure of Boc-diphenylalanine hydrogels was also characterized by circular dichroism (CD) (Figure 6 and Figure 7) [46,47]. The gels were prepared in a vial and directly added to a cylindrical 0.05 mm quartz cuvette after the addition of HCl. The CD spectrum of Boc-α(*S*)Phe-α(*S*)Phe-OH showed no signal at t0, but a negative peak appeared already after 1 h at 219 nm and increased over time, in agreement with the long gelation time of the α-diphenylalanine analogue (Figure 6).

The signal observed at 219 nm can be attributed to the overlapping of the π–π* phenyl side chain transition and the hydrogen bond network involving the carbamate functionality [39]. The secondary structure of Boc-α(*S*)Phe-α(*S*)Phe-OH reached at equilibrium is similar to that reported for other peptide hydrogels bearing diphenylalanine motifs displaying a β-sheet structure [47]. Unfortunately, the instability of the Boc-α(*S*)Phe-α(*S*)Phe-OH gels containing 0.025 wt% ox-CNTs or GO hampered their analysis by CD. In the case of the Boc-β^3^(*R*)Phe-β^3^(*R*)Phe-OH hydrogel, the negative peak at 219 nm, whose intensity remained constant over a period of 30 min, was already observed at t0 (Figure 7). This result confirmed the instantaneous gelation and the stability of the Boc-β^3^(*R*)Phe-β^3^(*R*)Phe-OH hydrogel after the addition of HCl. We observed the same signal and gelation kinetics in the presence of ox-CNTs and GO. In the case of Boc-γ^4^(*R*)Phe-γ^4^(*R*)Phe-OH, a signal was observed after 10 min, which was different from the two other peptides analogues with a positive peak at 220 nm and a negative peak at 193 nm [22]. The signal remained unchanged after 30 min and could be attributed to the π-π* phenyl side chain transition and hydrogen bond network involving the carbamate functionality. A similar trend was also observed in the presence of ox-CNTs and GO.

The CD results confirmed the self-assembly of the dipeptides during the gelation and the faster gelation process for the Boc-β^3^(*R*)Phe-β^3^(*R*)Phe-OH and Boc-γ^4^(*R*)Phe-γ^4^(*R*)Phe-OH hydrogels compared to the Boc-α(*S*)Phe-α(*S*)Phe-OH. We also confirmed that the presence of 0.025 wt% of ox-CNTs or GO did not impact the gelation process.

### 3.6. Drug Loading and Release

Short-peptide hydrogels, due to their tunable physical properties and controllable stability, can be considered promising platforms for drug delivery applications [48,49]. In this context, we studied the incorporation of l-ascorbic acid as model hydrophilic drug into the Boc-diphenylalanine hybrid hydrogels at a concentration of 0.7 mg·mL^−1^ [39]. To form the native hydrogels, the dipeptides were dissolved in a basic solution of the drug. After fast sonication and heating, a few HCl drops were added to induce the gelation. The presence of l-ascorbic acid did not impact the gelation properties of the three Boc-diphenylalanine analogues. To form the hybrid hydrogels, the carbon nanomaterials (ox-CNTs or GO) were dissolved into the basic solution containing the dipeptide. Then, the drug was added before changing the pH to acid to trigger the gelation. Almost all dipeptides formed hydrogels, except Boc-γ^4^(*R*)Phe-γ^4^(*R*)Phe-OH in the presence of l-ascorbic acid and ox-CNTs.

**Figure 8:**
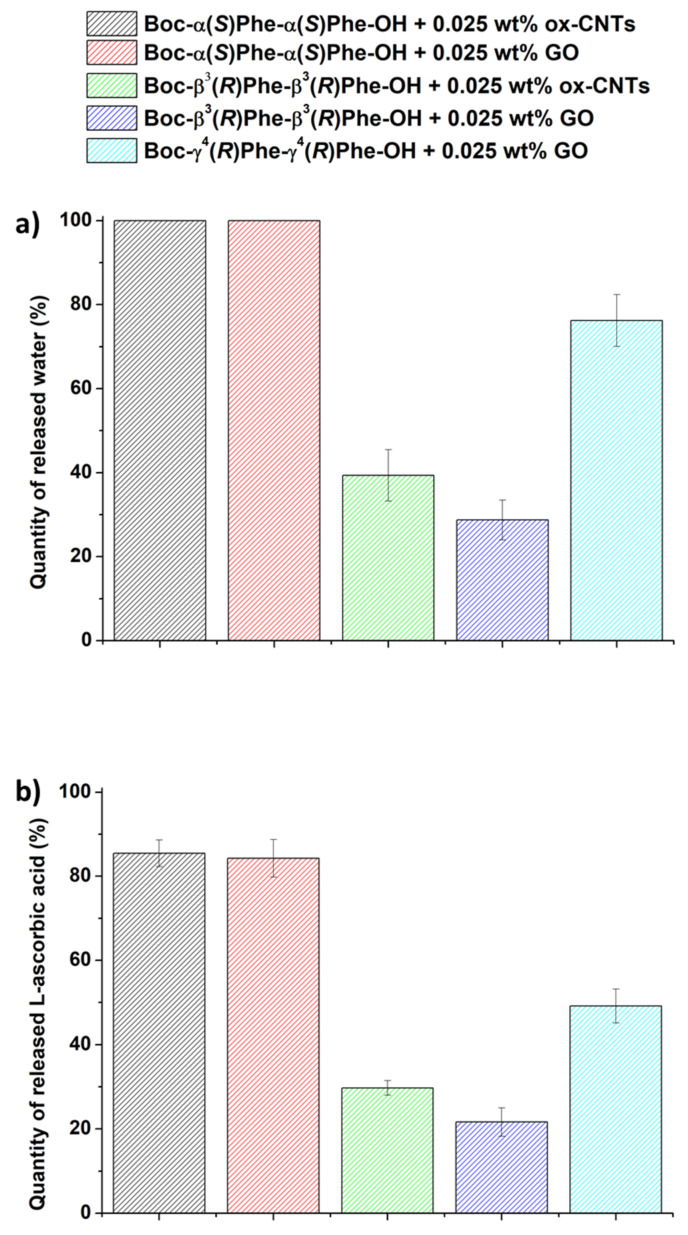
Release of (**a**) water (n = 3) and (**b**) l-ascorbic acid (n = 3) from the hybrid gels under NIR light irradiation.

We investigated the photothermal properties of the drug-loaded hydrogels under NIR light irradiation, as well as the water and drug release (Figure 8). For this purpose, we used an infrared laser at a power of 2 W·cm^−^^2^ at a distance of 3 cm from the top of the sample during 10 min. The temperature was measured every minute during the irradiation. After 10-min irradiation, the water released from the sample was withdrawn to measure the volume released. The solution was analyzed by HPLC to determine the amount of drug released.

Both Boc-α(*S*)Phe-α(*S*)Phe-OH hybrid gels were fully degraded after 10 min of irradiation, resulting in 100% water release (Figure 8a). Boc-γ^4^(*R*)Phe-γ^4^(*R*)Phe-OH + GO also showed a high volume of water released (76%). Both hybrid gels made of Boc-β^3^(*R*)Phe-β^3^(*R*)Phe-OH released a low volume of water of 29% and 39% in the presence of GO and ox-CNTs, respectively. The amount of drug released was determined by HPLC and it was consistent with the amount of water released. Both Boc-α(*S*)Phe-α(*S*)Phe-OH hybrid fully degraded gels released all of the l-ascorbic acid loaded in the gel after 10 min of irradiation. The Boc-γ^4^(*R*)Phe-γ^4^(*R*)Phe-OH + GO hydrogel showed a higher amount of released l-ascorbic acid (49%) compared to the Boc-β^3^(*R*)Phe-β^3^(*R*)Phe-OH hybrid gels (22% and 30% in the presence of GO and ox-CNTs, respectively). Interestingly, the gel Boc-β^3^(*R*)Phe-β^3^(*R*)Phe-OH + GO showed the highest temperature increase (Figure 4), but the lowest release of the drug and water compared to the other hydrogels. This behavior may be due to the fast gelation of Boc-β^3^(*R*)Phe-β^3^(*R*)Phe-OH giving a better distribution of the carbon nanomaterials within the hydrogel matrix, thus resulting in the highest temperature increase. The interactions between the drug and the Boc-β^3^(*R*)Phe-β^3^(*R*)Phe-OH and/or the carbon nanomaterials are probably very strong and might hamper an efficient release of the drug. The highest water and drug release obtained for the hybrid α-hydrogels are due to their lowest stability compared to the β- and γ-analogue. With its homogeneity, high stability over time, and high drug release under NIR light irradiation, the Boc-γ^4^(*R*)Phe-γ^4^(*R*)Phe-OH + GO hydrogel is the most suitable gel for controlled drug release applications. l-Ascorbic acid has been also incorporated in other types of hydrogels, mainly constituted of pH- or thermo-responsive (bio)polymers [50,51]. The release of the drug was spontaneous at 37 °C [51], or triggered by changing pH [50], but it reached lower percentages in comparison to our photoresponsive hydrogels.

## 4. Conclusions

In this work, we investigated the self-assembly behavior of Boc-α-diphenylalanine and Boc-α-dityrosine, as well as their β- and γ-homologues in water. The Boc-diphenylalanine analogues were able to self-organize in nanospheres (Boc-α-Phe-α-Phe-OH) or nanofibers (Boc-β^3^(*R*)Phe-β^3^(*R*)Phe-OH and Boc-γ^4^(*R*)Phe-γ^4^(*R*)Phe-OH), whereas Boc-dityrosine analogues showed the formation of aggregates with no defined morphology. These observations illustrate the influence of the hydroxyl group of the tyrosine lateral chain in the self-assembly process. The pH-switch protocol was the most efficient method to form hydrogels for the three Boc-diphenylalanine peptides, whereas the Boc-dityrosine analogues were not able to jellify. l-Ascorbic acid and ox-CNTs or GO were incorporated into the hydrogels without impacting the gelation process. Upon NIR light irradiation, l-ascorbic acid was released at a high concentration due to the destabilization of the gel structure caused by the photothermal effect of the carbon nanomaterials. These results suggest how hydrogels based on short homopeptides containing light-responsive nanomaterials can be considered promising systems for controlled on-demand drug release applications. Further studies will consist of evaluating their potential as injectable hydrogels for subcutaneous applications using skin and mice models.

## Figures and Tables

**Table 1:** Summary of the hydrogelation tests performed for Boc-α(*S*)Phe-α(*S*)Phe-OH, Boc-β^3^(*R*)Phe-β^3^(*R*)Phe-OH, and Boc-γ^4^(*R*)Phe-γ^4^(*R*)Phe-OH.

	Boc-α(*S*)Phe-α(*S*)Phe-OH			Boc-β^3^(*R*)Phe-β^3^(*R*)Phe-OH			Boc-γ^4^(*R*)Phe-γ^4^(*R*)Phe-OH		
	Aspect	Gelation duration	Stability in time	Aspect	Gelation duration	Stability in time	Aspect	Gelation duration	Stability in time
T = 4 °C	Viscous liquid	/	/	Heterogenous gel	13 h	yes	**Homogenous gel**	<5 min	yes
Ambient						no			
T = 37 °C	**Homogenous gel**	15 h	no						
pH = 4	Heterogenous gel	2 h	no	Heterogeneous gel	24 h	yes	**Homogenous gel**	<5 min	yes
pH = 7		15 h		Heterogenous viscous liquid	/	/			
pH = 11		15 h			/	/	Viscous liquid	/	/
2.45 mM	**Homogenous gel**	21 h	no	Heterogenous liquid	/	/	Heterogenous liquid	/	/
9.8 mM	Heterogenous gel	15 h	no	Heterogeneous gel	1 h	yes	**Homogenous gel**	<5 min	yes
2% HFIP	Heterogenous liquid	/	/	Heterogenous liquid	/	/	Heterogenous liquid	/	/
2% MeOH	Heterogenous liquid	/	/	Heterogenous gel	24 h	yes	Heterogenous liquid	/	/
5% DMSO	/	/	/	Heterogenous gel	1 h	no	/	/	/
10% DMSO	/	/	/	Heterogenous gel	5 min	yes	/	/	/
NaCl	Heterogenous liquid	/	/	Heterogenous liquid	/	/	Heterogenous liquid	/	/
CaCl_2_		/	/		/	/		/	
pH switch	**Homogenous gel**	7 h	1 day	**Homogenous gel**	<1 min	yes	**Homogenous gel**	<1 h	yes

## Data Availability

Data are available from the authors upon request.

## Supplementary Materials

The following supporting information can be downloaded at: https://www.mdpi.com/article/10.3390/nano12101643/s1. Schematic representation of the steps followed to make the different gels; digital pictures of the hydrogel formation tests; diameter distribution of the fibrils in the hydrogels.
