# Supplementary material for: Aromatic Dipeptide Homologue-Based Hydrogels for Photocontrolled Drug Release
Source: Nanomaterials (Basel). 2022 May 11;12(10):1643. doi: 10.3390/nano12101643 (PMC9143549; doi:10.3390/nano12101643)
Supplement: Supplementary file 1 [file nanomaterials-12-01643-s001.zip › nanomaterials-1652572-supplementary.pdf]

nanomaterials

Supplementary Information

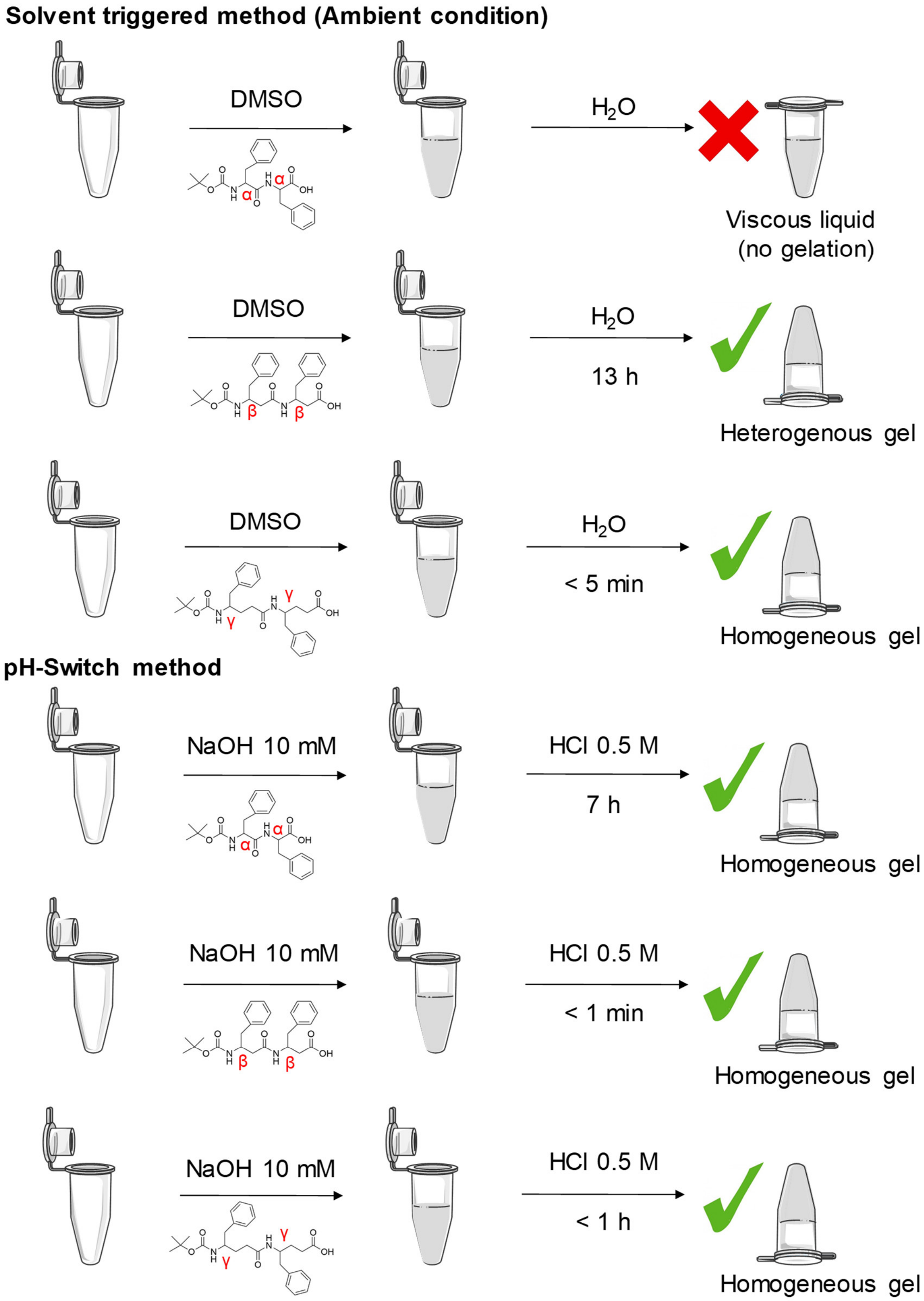

Figure S1. Schematic representation of the steps followed to make the different gels.

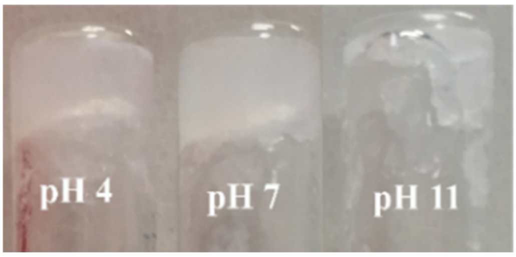

Figure S2. Assessment of the formation of hydrogels using the vial inversion test for Boc-γ[4](*R*)Phe-γ[4](*R*)Phe-OH (4.9 mM, 2% DMSO/H_2O) at different pH.

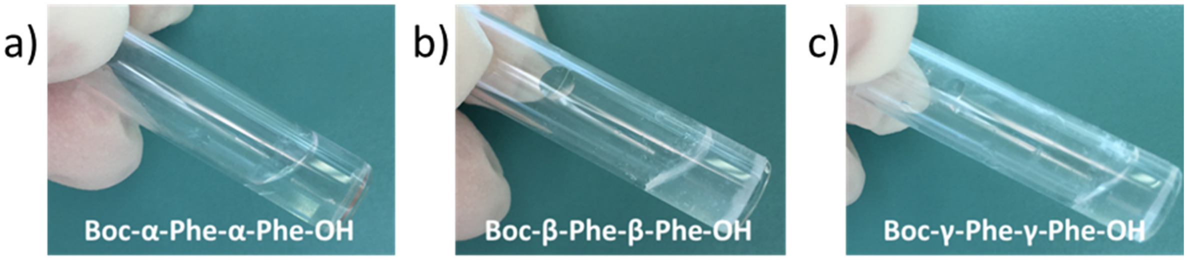

Figure S3. Assessment of the formation of hydrogels in 2% HFIP/H_2O using 4.9 mM of a) Boc-α(*S*)Phe-α(*S*)Phe-OH, b) Boc-β[3](*R*)Phe-β[3](*R*)Phe-OH, and c) Boc-γ[4](*R*)Phe-γ[4](*R*)Phe-OH.

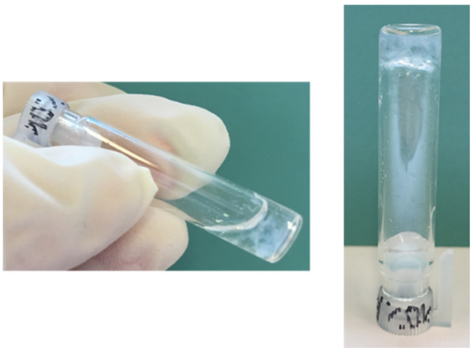

Figure S4. Hydrogel test formation of 4.9 mM Boc-β[3](*R*)Phe-β[3](*R*)Phe-OH in 10% DMSO/H_2O at t0 and after 5 min.

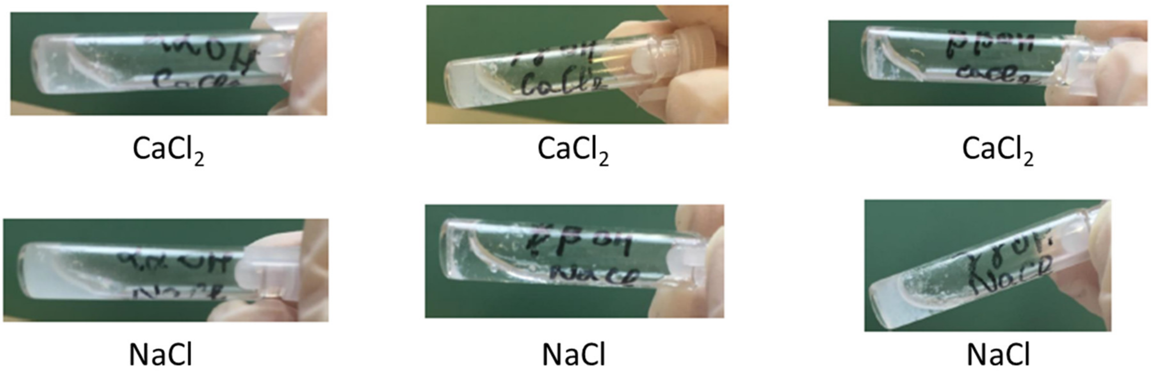

Figure S5. Hydrogel test formation of 4.9 mM diphenylalanine analogues in 2% DMSO/H_2O in the presence of 25 mM $CaCl_2$ or NaCl (from left to right: Boc-α(*S*)Phe-α(*S*)Phe-OH, Boc-β[3](*R*)Phe-β[3](*R*)Phe-OH, and Boc-γ[4](*R*)Phe-γ[4](*R*)Phe-OH).

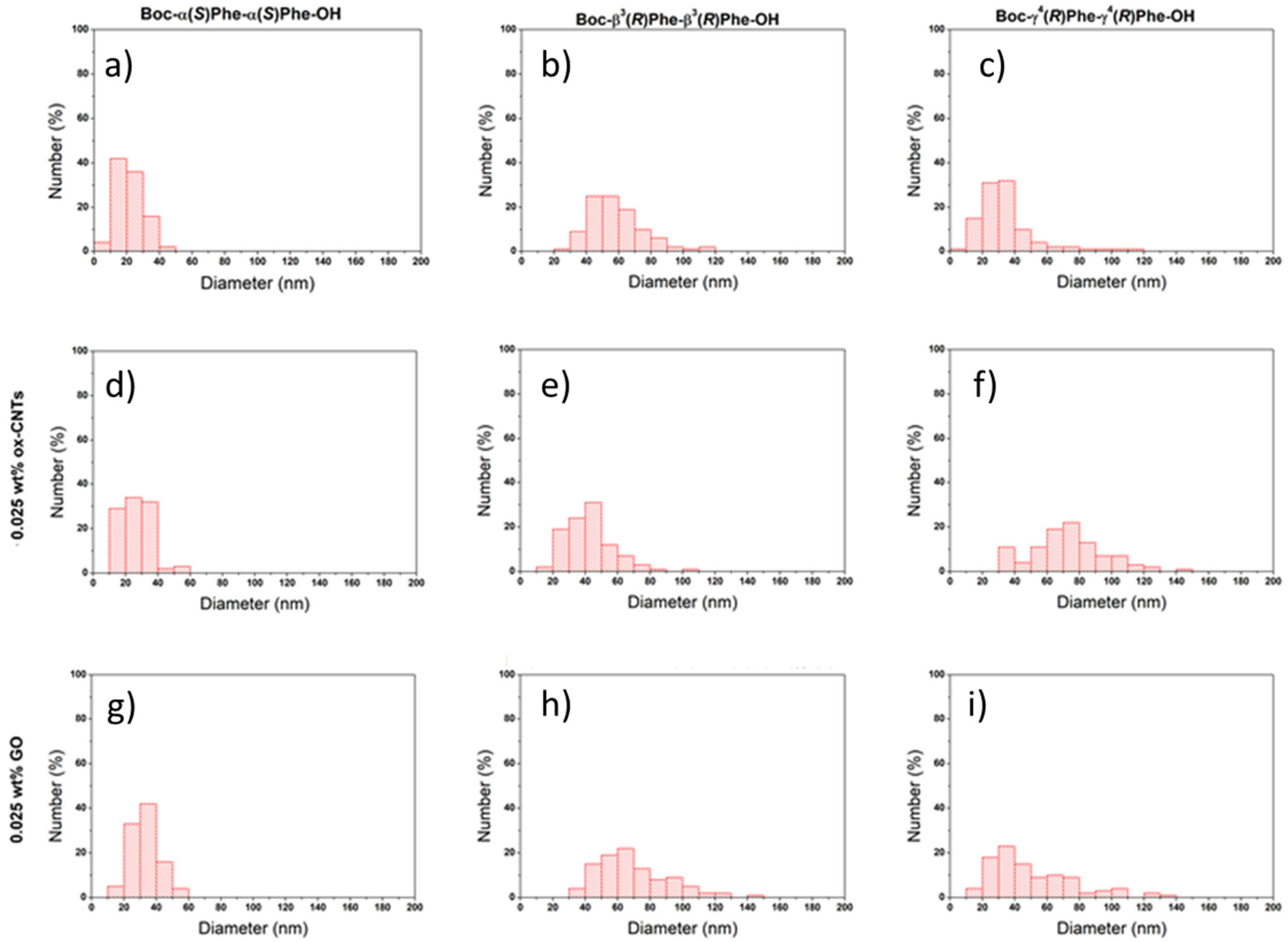

Figure S6. Diameter distribution of the fibrils of the native and hybrid hydrogels (n=100).
